# Recent Antiretroviral Therapy Initiation Is Associated With Increased Mortality Risk in HIV–associated Cryptococcal Meningitis: An Analysis of Clinical Trial Data From Africa

**DOI:** 10.1093/cid/ciae586

**Published:** 2025-01-15

**Authors:** Melanie Moyo, David S Lawrence, James Jafali, Síle F Molloy, Johnstone Kumwenda, Cecilia Kanyama, Mina C Hosseinipour, Chiratidzo E Ndhlovu, Mosepele Mosepele, David B Meya, Joshua Rhein, David R Boulware, Conrad Muzoora, Rishi K Gupta, Thomas H A Samuels, Nabila Youssouf, Timothée Boyer Chammard, Olivier Lortholary, Charlotte Schutz, Graeme Meintjes, Henry C Mwandumba, Thomas S Harrison, Joseph N Jarvis

**Affiliations:** Malawi-Liverpool-Wellcome Programme, Blantyre, Malawi; Department of Medicine, Kamuzu University of Health Sciences, Blantyre, Malawi; Department of Clinical Research, Faculty of Infectious and Tropical Diseases, London School of Hygiene and Tropical Medicine, London, United Kingdom; Botswana-Harvard Health Partnership, Gaborone, Botswana; Malawi-Liverpool-Wellcome Programme, Blantyre, Malawi; Centre for Global Health, Institute of Infection and Immunity, St George's University of London, London, United Kingdom; Department of Medicine, Kamuzu University of Health Sciences, Blantyre, Malawi; University of North Carolina Project, Kamuzu Central Hospital, Lilongwe, Malawi; University of North Carolina Project, Kamuzu Central Hospital, Lilongwe, Malawi; Department of Medicine, University of North Carolina at Chapel Hill School of Medicine, Chapel Hill North Carolina USA; Department of Internal Medicine, Faculty of Medicine and Health Sciences, University of Zimbabwe, Harare, Zimbabwe; Botswana-Harvard Health Partnership, Gaborone, Botswana; Department of Internal Medicine, Faculty of Medicine, University of Botswana, Gaborone, Botswana; Infectious Diseases Institute, College of Health Sciences, Makerere University, Kampala, Uganda; Infectious Diseases Institute, College of Health Sciences, Makerere University, Kampala, Uganda; Division of Infectious Diseases & International Medicine, Department of Medicine, University of Minnesota, Minneapolis, MN, USA; Division of Infectious Diseases & International Medicine, Department of Medicine, University of Minnesota, Minneapolis, MN, USA; Department of Medicine, Mbarara University of Science and Technology, Mbarara, Uganda; Institute for Global Health, University College London, London, United Kingdom; Institute for Global Health, University College London, London, United Kingdom; Department of Clinical Research, Faculty of Infectious and Tropical Diseases, London School of Hygiene and Tropical Medicine, London, United Kingdom; Botswana-Harvard Health Partnership, Gaborone, Botswana; Necker Pasteur Center for Infectious Diseases and Tropical Medicine, Hôpital Necker Enfants malades, AP-HP, IHU Imagine, Université de Paris, Paris, France; Molecular Mycology Unit and National Reference Centre for Invasive Mycoses, UMR2000, CNRS, Institut Pasteur, Paris, France; Necker Pasteur Center for Infectious Diseases and Tropical Medicine, Hôpital Necker Enfants malades, AP-HP, IHU Imagine, Université de Paris, Paris, France; Molecular Mycology Unit and National Reference Centre for Invasive Mycoses, UMR2000, CNRS, Institut Pasteur, Paris, France; Wellcome Centre for Infectious Diseases Research in Africa (CIDRI-Africa), Institute of Infectious Disease and Molecular Medicine, University of Cape Town, Cape Town, South Africa; Department of Medicine, University of Cape Town, Cape Town, South Africa; Wellcome Centre for Infectious Diseases Research in Africa (CIDRI-Africa), Institute of Infectious Disease and Molecular Medicine, University of Cape Town, Cape Town, South Africa; Department of Medicine, University of Cape Town, Cape Town, South Africa; Blizard Institute, Queen Mary University of London, London, United Kingdom; Malawi-Liverpool-Wellcome Programme, Blantyre, Malawi; Department of Medicine, Kamuzu University of Health Sciences, Blantyre, Malawi; Department of Clinical Sciences, Liverpool School of Tropical Medicine, Liverpool, United Kingdom; Centre for Global Health, Institute of Infection and Immunity, St George's University of London, London, United Kingdom; Department of Clinical Research, Faculty of Infectious and Tropical Diseases, London School of Hygiene and Tropical Medicine, London, United Kingdom; Botswana-Harvard Health Partnership, Gaborone, Botswana

**Keywords:** HIV, AIDS, cryptococcal meningitis, cryptococcosis, antiretroviral therapy

## Abstract

**Background:**

More than half of people diagnosed with human immunodeficiency virus–associated cryptococcal meningitis are antiretroviral therapy (ART)-experienced. The impact of recent ART initiation (≤14 days) on outcomes from cryptococcal meningitis, and how to optimally manage ART in this patient population, are unknown.

**Methods:**

We analyzed data from the recent Ambisome Therapy Induction Optimisation (AMBITION) trial to (1) examine whether patients diagnosed with cryptococcal meningitis within 14 days of ART initiation are at higher risk of mortality and (2) determine the impact of ART interruption at diagnosis of cryptococcal meningitis. Combined data from the AMBITION trial and the earlier Antifungal Combinations for Treatment of Cryptococcal Meningitis in Africa trial were analyzed to describe baseline characteristics of patients according to ART status.

**Results:**

Among the 810 AMBITION participants, adjusted 2-week mortality risk was 20.8% (95% confidence interval [CI]: 11.5–30.2; 26/120) in those on ART for 14 days or less at presentation, 10.4% (95% CI: 3.6–17.2; 18/130) on ART for >2 weeks to 2 months, 7.1% (95% CI: 0–14.9; 7/92) on ART for >2 months to 6 months, and 13.0% (95% CI: 8.5–17.6; 50/307) in those on ART for more than 6 months compared to 12.4% (95% CI: 9.2–15.5; 111/707) among individuals not on ART. In the combined dataset, baseline fungal burdens were lower and baseline CD4 counts were higher with increasing ART duration. Among individuals on ART for ≤14 days at presentation, 2-week mortality was 35% (8/23) in those continuing ART versus 14% (7/49) in those discontinuing ART.

**Conclusions:**

Mortality from cryptococcal meningitis was higher in recent ART initiators. ART interruption in this group may lead to improved outcomes.

Cryptococcal meningitis causes up to 19% of all AIDS-related deaths globally [1]. In recent studies of human immunodeficiency virus (HIV)-associated cryptococcal meningitis, more than half of participants were antiretroviral therapy (ART)-experienced (currently receiving or previously received ART) when developing meningitis [2,3]. The evidence base for ART management among ART-naïve individuals presenting with cryptococcal meningitis is clear, with a causal association between early ART initiation following antifungal treatment and excess mortality; the COAT trial showed that ART initiation should be delayed for 4–6 weeks in ART-naïve individuals presenting with cryptococcal meningitis [4, 5]. However, the implications of being on ART at the time of diagnosis with cryptococcal meningitis, and the optimal ART management strategies in individuals presenting with cryptococcal meningitis after ART initiation, are less well understood.

Studies have consistently shown no overall mortality difference between ART-naïve and ART-experienced individuals with cryptococcal meningitis [3, 6, 7]. But a secondary analysis from the Adjunctive Sertraline for Treatment of Cryptococcal Meningitis trial suggested an increased risk of mortality in people with HIV who presented with cryptococcal meningitis within 14 days of initiating ART compared with those on ART for longer (hazard ratio [HR], 1.90 [1.2–2.9]; *P* < .01) [8], with similar finding in the Antifungal Combinations for Treatment of Cryptococcal Meningitis in Africa (ACTA) trial (HR, 1.70 [0.85–3.39]) [9]. Individuals presenting with cryptococcal meningitis after initiating ART within the previous 14 days are highly likely to have had undiagnosed cryptococcal meningitis at the time of ART initiation. It has been hypothesized that these recent ART initiators may have increased mortality risk because of excessive immune responses induced by ART initiation in the context of active cryptococcal meningitis [8] similar to that seen with early ART initiation among ART-naive individuals with cryptococcal meningitis [4, 5], and that temporary discontinuation of ART may improve outcomes.

In addition to the group of individuals started on ART within the 14 days before their diagnosis of cryptococcal meningitis, with probable active cryptococcal meningitis at the time of ART start, a further proportion diagnosed with cryptococcal meningitis may have had asymptomatic or early subclinical cryptococcal infection at the time of ART initiation and present with “unmasking” immune reconstitution inflammatory syndrome (IRIS), typically within 2 months of ART initiation. There are also individuals taking effective ART but who have not yet achieved significant reconstitution of CD4 counts, leaving them vulnerable to cryptococcal disease. Finally, a proportion have been on ART for longer durations but may have virological nonsuppression due to challenges with ART adherence or HIV drug resistance [2, 3, 8, 10]. The population developing cryptococcal meningitis after ART initiation is therefore a heterogenous group who need different ART management strategies.

To address these evidence gaps, we performed a secondary analysis of Ambisome Therapy Induction Optimisation (AMBITION) trial data [3] and another analysis combining AMBITION data with the preceding ACTA trial [2] to (1) examine whether patients who are diagnosed with cryptococcal meningitis within 14 days of ART initiation are at higher risk of mortality when compared to those not on ART or on ART for longer durations; (2) to describe the baseline characteristics of individuals presenting with cryptococcal meningitis according to their ART status; and (3) to determine whether interrupting ART at diagnosis of cryptococcal meningitis in recent ART initiators improves clinical outcomes.

## METHODS

### Study Population

For our initial analysis, the study population comprised all AMBITION trial participants. ART status and duration data were captured for all participants. A standardized ART management approach including ART discontinuation in those who had initiated ART in the 14 days preceding trial enrolment was recommended [11], but ART management was at the discretion of the attending clinicians.

To increase study power, participants from the AMBITION and the preceding ACTA trials, which had comparable eligibility criteria, were combined in a second analysis to describe baseline associations with ART status and perform a secondary analysis of outcomes. ACTA data were excluded from the initial analysis of outcomes for 2 reasons: (1) ART timing data were missing for 34% of individuals; these data were unlikely to be missing at random with more missing data in those who were very unwell at admission and died early, and for all patients who were on ART in the ACTA trial continuation was recommended and (2) ACTA had 5 treatment arms with significantly differing mortality rates.

### Study Design and Data Analysis

The main exposure was time on ART prior to cryptococcal meningitis diagnosis. Participants were grouped according to ART status into those “on ART” and those “not taking ART,” which included both ART-naïve patients and those who had disengaged from ART (defined as having not taken ART for at least 60 consecutive days). Participants on ART were classified according to the duration of effective ART (ie, period from ART introduction, reintroduction, or ART regimen switch). Because there are no standardized criteria, categories were defined a priori based on expert opinion as follows:

- On ART ≤2 weeks at presentation with cryptococcal meningitis; 14 days was the median duration of headache reported by trial participants in the AMBITION trial [3]; therefore, individuals in this group were likely to have had cryptococcal meningitis at the time of ART initiation. This categorization enabled comparison with published literature [2, 8].
- On ART from >2 weeks to ≤2 months; individuals who were likely to have had subclinical cryptococcal infection at the time of ART initiation based on published literature showing that blood cryptococcal antigen is detectable at least 35 days before development of cryptococcal meningitis [12], including those with an “unmasking” IRIS that typically occurs within 2 months of ART initiation [13].
- On ART for >2 months to ≤6 months; selected as a group “established” on ART, based on median times from ART initiation to virological suppression, but who have not yet achieved significant reconstitution of CD4 counts [14].
- On ART >6 months; individuals likely to be immunosuppressed due to difficulties with adherence to ART or HIV drug resistance.

All-cause mortality within 2 weeks was the primary outcome, with 10-week mortality as a secondary outcome. We hypothesized that participants diagnosed with cryptococcal meningitis within 14 days of ART initiation, reinitiation or regimen switch would have higher all-cause mortality and that ART interruption reduces mortality risk in these patients by preventing pathological immune reconstitution reactions.

A generalized linear model with a binomial distribution and log link function was used to calculate the differences in mortality risk and risk ratios between ART groups. Mortality estimates were adjusted for the antifungal induction regimen. Age and sex were included in the model if they modified the risk ratio by more than 5%. Variables that may be on the causal pathway between ART status and outcome, including CD4 count, cerebrospinal fluid (CSF) white cell count, and CSF fungal burden, were not included in the adjusted models. ART discontinuation was added to a model as an interaction term to determine whether it modified the association between recent ART initiation and mortality risk. As an indicative sample size, 118 individuals per group would give an 80% power to detect a 15% absolute 2-week mortality risk increase from 15% to 30% at an alpha of 0.05.

To describe the dynamic relationship between time on ART and mortality risk, in those who had recently started ART, univariable logistic regression models were constructed with days on ART as the single predictor (using restricted cubic splines with 3 knots) and mortality as the outcome (2-week and 10-week), restricted to participants on ART for up to 6 months.

Analyses were conducted using R and Stata version 17 (College Station, TX).

### Ethical Considerations

Both trials were approved by the London School of Hygiene and Tropical Medicine Ethics Committee. Participating sites obtained approvals from local regulatory and ethics authorities.

## RESULTS

### Study Population

We analyzed data from 810 AMBITION participants enrolled between 2018 and 2021. The median age was 37 years (interquartile range [IQR] 32–43); 491/810 (60.6%) were male; and the median CD4 count was 27 cells/µL (IQR 10–58); 398/810 (49%) were currently taking ART (Table 1). ART duration data were available for all individuals taking ART, with a median duration of 9 months (269 days, IQR 25–1952 days); 72/398 (18%) had initiated ART ≤14 days before presentation with cryptococcal meningitis, 77/398 (19%) had been on ART between >14 days and 2 months, 42/398 (11%) between >2 months and 6 months, and 207/398 (52%) had been on ART for >6 months.

**Table 1.** Baseline Characteristics of Study Participants

Characteristic		AMBITION Trial N = 810^a^	ACTA Trial N = 674^b^	Combined Cohort N = 1484
Treatment groups^c^		1. Liposomal amphotericin B 10 mg/kg on d 1 plus fluconazole and 5FC for 14 d 2. Amphotericin B deoxycholate 1 mg/kg/day plus 5FC for 7 d followed by fluconazole for 7 d	1. Amphotericin B deoxycholate 1 mg/kg/day plus 5FC for 7 d followed by fluconazole for 7 d 2. Amphotericin B deoxycholate 1 mg/kg/day plus 5FC for 14 d 3. Amphotericin B deoxycholate 1 mg/kg/day plus fluconazole for 7 d followed by fluconazole for 7 d 4. Amphotericin B deoxycholate 1 mg/kg/day plus fluconazole for 14 d 5. Fluconazole plus 5FC for 14 d	…
Age, y		37 (32–43)	37 (32–43)	37 (32–43)
Male		60.6% (491)	57.3% (386)	59.1% (877)
CD4 cell count, cells/µL		27 (10–58)	26 (10–63)	27 (10–62)
Baseline CSF fungal burden, log_10_ colony forming units/mL CSF		4.66 (2.6–5.6)	4.98 (3.7–5.8)	4.79 (3.1–5.7)
Abnormal mental status with Glasgow Coma Score <15		28.5% (231)	24.0% (162)	26.5% (393)
ART status				
Not on ART	Overall	50.9% (412)	43.8% (295)^d^	47.6% (707)
	ART naïve	70.1% (289)	100% (295)	82.6% (584)
	Interrupted ART	29.9% (123)	0	17.4% (123)
On ART	Overall	49.1% (398)	56.2% (379/674)^d^	52.4% (777/1484)
	≤14 d	18% (72/398)	19% (48/251)	18.5% (120/649)
	>14 d ≤2 m	19% (77/398)	21% (53/251)	20.0% (130/649)
	>2 m ≤6 m	11% (42/398)	20% (50/251)	14.2% (92/649)
	>6 m	52% (207/398)	40% (100/251)	47.3% (307/649)

Values are median (IQR) or % (n).

Abbreviations: ACTA, Antifungal Combinations for Treatment of Cryptococcal Meningitis in Africa; AMBITION; AmBisome Therapy Induction Optimisation; ART, antiretroviral therapy; CFU, colony forming units; CSF, cerebrospinal fluid; 5FC, flucytosine; IQR, interquartile range.

^a^The AMBITION trial included 844 participants, 814 of whom were included in the intention to treat analysis after late exclusions. Of these, 4 participants withdrew consent for further analyses, leaving the 810 individuals included here.

^b^The ACTA trial included 674 participants. ART timing data were only available for 251 of the 379 participants on ART. Analysis of ART timing and mortality was therefore restricted to AMBITION trial participants.

^c^The dose of fluconazole used was 1200 mg/day in all trial arms. The dose of flucytosine used was 100 mg/kg/day in 4 divided doses in all trial arms. All individuals received standard consolidation and maintenance therapy with fluconazole 800 mg/day for 8 wk followed by 200 mg/day until CD4 counts were above 200 cells/µL.

^d^Data regarding prior ART exposure in those not on ART in the ACTA trial were not available. ART timing data were missing for 128 participants on ART in the ACTA trial.

**Figure 1.**
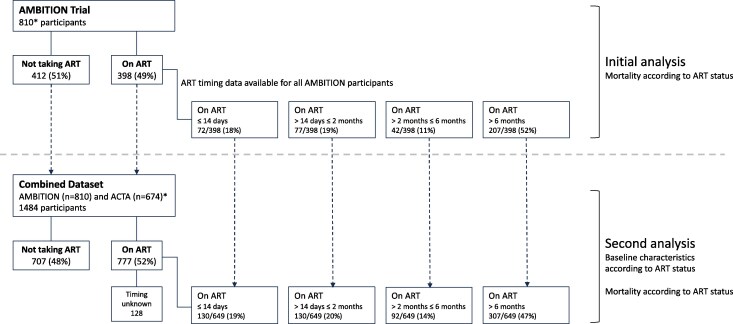
Classification of antiretroviral therapy (ART) status at time of presentation with cryptococcal meningitis in the Ambisome Therapy Induction Optimisation (AMBITION) trial and combined AMBITION and Antifungal Combinations for Treatment of Cryptococcal Meningitis in Africa (ACTA) trial datasets. Data were analyzed from 810 AMBITION participants. ART duration data were available for all individuals taking ART. A total of 674 ACTA participants with complete outcome data were added to a combined secondary analysis, giving a total of 1484 individuals. ACTA data were excluded from primary mortality analysis as ART timing data were missing for 34% of individuals; the combined population was used to describe baseline characteristics according to ART status and for secondary analysis of outcomes. *The AMBITION trial randomized 844 participants, 30 of whom met late exclusion criteria and were not included, and further 4 who did not give consent for additional analysis, leaving 810 individuals for this study. The ACTA trial included 674 participants with complete outcome data who were added into the combined dataset; however, although it was known who was taking ART at baseline in the ACTA trial, ART timing data were missing for 128 participants of the 379 on ART.

A total of 674 ACTA participants with complete outcome data were included in the combined second analysis; enrolled between 2013 and 2016. Baseline characteristics were similar to those enrolled on the AMBITION trial (Table 1). Combining the 2 studies gave a total of 1484 individuals; this combined population was used to describe baseline characteristics according to ART status and for second supportive analysis of outcomes (Figure 1).

### Baseline Characteristics According to ART Status

Participants presenting with cryptococcal meningitis while taking ART had similar demographics to those not on ART but had higher baseline CD4 counts and lower baseline fungal burdens in both the AMBITION study and combined dataset (Table 2). There were no significant differences in antifungal treatment allocation between those taking ART and those not on ART. In the combined dataset, CD4 counts and baseline fungal burdens differed significantly (*P* < .001) by time on ART; individuals on ART for >6 months had a median CD4 count of 21 cells/µL, comparable to individuals not on ART whose median CD4 count was 23 cells/µL, whereas those on ART <14 days, > 2 weeks to 2 months, and >2 months to 6 months, had CD4 counts of 41 cells/µL, 47 cells/µL, and 55 cells/µL, respectively. Those on ART for >6 months had high fungal burdens, comparable to those not on ART; among those on ART for up to 6 months, baseline fungal burdens declined with increasing ART duration (Table 2). Of those who were on ART for >6 months, 93/307 (30%) reported poor ART adherence having missed a median of 8 doses in the past month (IQR 4−15), compared to 27/342 (8%) on ART for less than 6 months; viral load results were available for 216 of the 307 individuals on ART for >6 months of whom 188/216 (87%) had detectable viral loads.

**Table 2.** Baseline Characteristic of Patients Presenting With Cryptococcal Meningitis According to ART Status (A) in the AMBITION Study and (B) in the Combined AMBITION and ACTA Datasets

		Not on ART	On ART				*P*
Variable	Median (IQR) % (n)		≤14 d	>14 d–2 m	>2 m–6 m	>6 m	
A. AMBITION Study Data							
Age (y)	**…**	37 (32–44)	38 (31–44)	38 (32–43)	36 (33–43)	36 (30–41)	.13
Sex (male, % n)	**…**	64% (262)	61% (44)	66% (51)	62% (26)	52% (108)	.07
CD4 cell count (cells/µL)	**…**	21 (9–44)	40 (20–66)	41 (25–89)	69 (37–107)	20 (9–63)	<.001
Baseline CSF fungal burden (log_10_ CFU/mL)	**…**	4.87 (3.3–5.7)	4.33 (2.3–5.2)	3.65 (1.8–5.1)	2.11 (1.2–3.5)	4.73 (2.6–5.7)	<.001
Abnormal mental status (Glasgow Coma Score <15)	**…**	30% (124)	39% (28)	27% (21)	26% (11)	23% (47)	.09
CSF white cell count (cells/µL)	**…**	5 (3–47)	5 (4–70)	15 (4–52)	35 (4–120)	6 (2–59)	.005
CSF protein (g/dL)	**…**	0.8 (0.4–1.4	0.9 (0.4–1.5)	1.1 (0.8–1.6)	1.1 (0.7–2.2)	0.8 (0.4–1.3)	.001
Symptom duration (d)	**…**	14 (7–21)	14 (7–21)^a^	14 (7–21)	14 (10–28)	14 (7–21)	.12
ART adherence (reporting good adherence)	**…**	*…*	92% (66)	90% (69)	88% (37)	69% (143)	<.001
Viral suppression^b^ (<50 copies/mL)	**…**	*…*	*…*	*…*	*…*	13% (23/184)	*–*
B. Combined AMBITION and ACTA Study Data							
Age (y)	…	37 (32–43)	39 (31–44)	39 (33–45)	36 (32–43)	36 (31–42)	.06
Sex (male, % n)	…	61% (433)	63% (75)	65% (84)	54% (50)	54% (166)	.11
CD4 cell count (cells/µL)	…	23 (9–48)	41 (21–73)	47 (22–86)	55 (30–93)	21 (9–63)	<.001
Baseline CSF fungal burden (log_10_ CFU/mL)	…	5.02 (3.8–5.7)	4.59 (2.6–5.5)	3.72 (2.0–5.1)	2.72 (1.2–4.4)	4.79 (3.0–5.7)	<.001
Abnormal mental status (Glasgow Coma Score <15)	…	28% (199)	36% (43)	25% (33)	23% (21)	22% (67)	.03
CSF white cell count (cells/µL)	…	4 (1–35)	4 (3–60)	7 (3–40)	6 (2–80)	5 (1–38)	.17
CSF protein (g/dL)	…	0.9 (0.5–1.5)	1.0 (0.5–1.5)	1.1 (0.8–1.8)	1.2 (0.8–2.2)	0.8 (0.4–1.4)	<.001
Symptom duration (d)	…	14 (7–21)	14 (7–21)^a^	14 (7–26)	14 (9–30)	14 (7–21)	.03
ART adherence (reporting good adherence)	…	…	95% (114)	92% (119)	89% (82)	70% (214)	<.001
Viral suppression^b^ (<50 copies/mL)	…	…	…	…	…	13% (28/216)	…

ART adherence was based on self-report, with those reporting no missed doses in the past month classified as reporting good adherence. *P* values were derived from Fisher exact test, chi-squared tests, or rank-sum tests as appropriate.

Abbreviations: ART, antiretroviral therapy; CSF, cerebrospinal fluid.

^a^91/115 (79%) of individuals on ART for ≤14 d had symptoms of cryptococcal meningitis at the time of ART initiation.

^b^Viral load testing is not performed until 6 m on therapy so viral load results were not available for most participants in the earlier timepoints. Viral load test results were available for 216/307 individuals on ART >6 m.

### Mortality Analyses

In the main analysis restricted to AMBITION trial data mortality was 13% (104/810) at 2 weeks and 27% (218/810) at 10 weeks. There were no significant mortality differences between participants taking ART and those not taking ART (Table 3); 2-week mortality was 12% (95% CI: 9.1–16.2) among those not on ART versus 13% (95% CI: 10.0–16.7) in the group on ART, and 10-week mortality was 26% (95% CI: 21.7–30.2) in those not on ART versus 28% (23.4–32.2) in those on ART.

**Table 3.** Mortality Outcomes Among AMBITION Participants With HIV-associated Cryptococcal Meningitis Stratified by ART Status at the Time of Presentation

ART Status^a^		2-week Mortality^b^			10-week Mortality^b^		
		Mortality risk (95% CI)	N	Risk ratio (95% CI)	Mortality risk (95% CI)	N	Risk ratio (95% CI)
A.							
Not on ART^c^		12.4% (9.1–16.2)	51/412	Base	26.0% (21.7–30.2)	107/412	Base
On ART	Overall	13.3% (10.0–16.7)	53/398	1.07 (0.8–1.5)	27.8% (23.4–32.2)	111/398	1.07 (0.9–1.3)
**…**	≤14 d	20.8% (11.5–30.2)	15/72	1.68 (1.0–2.8)	37.2% (26.1–48.3)	27/72	1.43 (1.0–2.0)
**…**	>14 d ≤2 m	10.4% (3.6–17.2)	8/77	0.84 (0.4–1.7)	22.0% (12.8–31.2)	17/77	0.85 (0.5–1.3)
**…**	>2 m ≤6 m	7.1% (0.0–14.9)	3/42	0.58 (0.2–1.8)	23.9% (11.0–37.8)	10/42	0.92 (0.5–1.6)
**…**	>6 m	13.0% (8.5–17.6)	27/207	1.05 (0.7–1.6)	27.5% (21.5–33.6)	57/207	1.05 (0.8–1.4)
B.							
On ART ≤14 d, ART continued		34.8% (15.4–57.2)	8/23	3.71 (1.8–7.5)	52.2% (30.6–73.1)	12/23	2.16 (1.4–3.4)
On ART ≤14 d, ART stopped		14.3% (5.9–27.2)	7/49	0.92 (0.4–2.1)	30.6% (18.3–45.4)	15/49	1.08 (0.6–1.8)

Abbreviations: ART: antiretroviral therapy; CI: confidence interval. N: raw numbers, numerator/denominator.

^a^Analysis restricted to the AMBITION study participants, for whom complete ART timing data were available. Given the missing ART timing data for ∼30% of ACTA trial participants it was felt that there was a high risk of bias; ART timing data were likely to be differentially missing in those who were most unwell with reduced consciousness or who died early during the course of treatment.

^b^Mortality risk derived from the generalized linear model adjusted for antifungal treatment group in the primary analysis presented in (A), with risk ratios derived using the group not on ART as the comparator. Age and sex were not included in the final models as they did not change the associations between exposure and outcome. For the secondary analysis examining the impact of ART discontinuation in those very recently initiated on ART-unadjusted mortality data are presented as the limited sample size precluded adjustment.

^c^Two-week mortality was the same in ART-naïve patients and ART defaulters (RR 0.98, *P* value .94), the 2 groups which were combined for this analysis.

**Figure 2.**
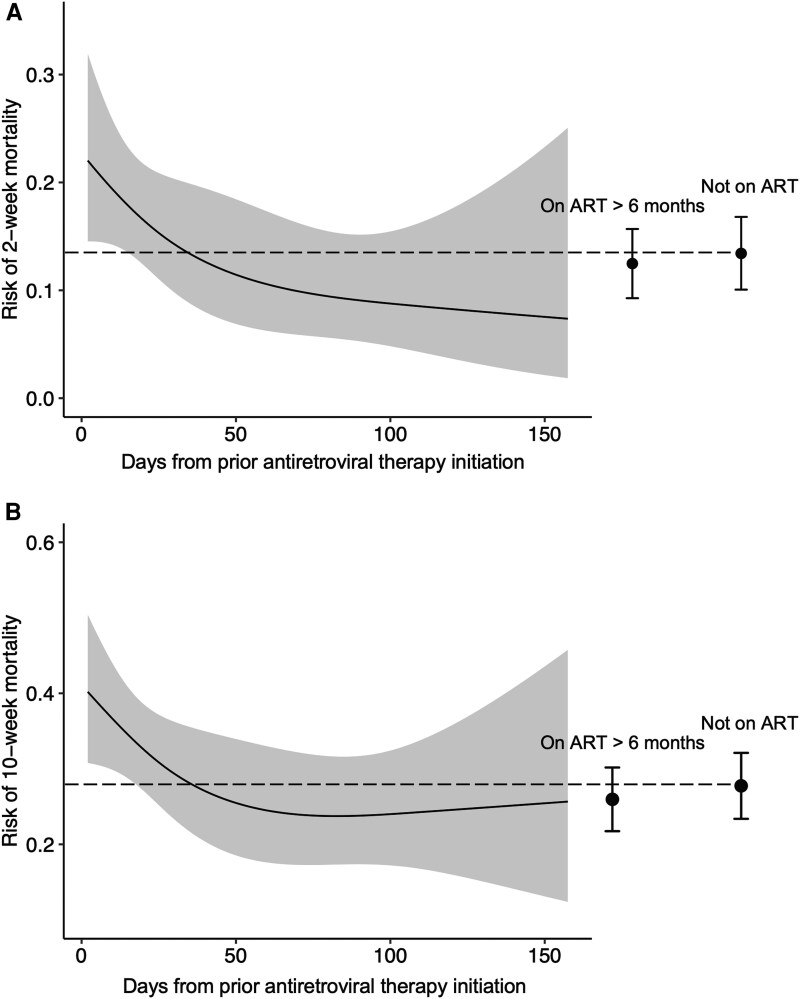
Mortality risk according to antiretroviral therapy (ART) status at the time of presentation with cryptococcal meningitis. The dynamic relationship between time on ART at cryptococcal meningitis presentation and mortality risk in the combined Ambisome Therapy Induction Optimisation (AMBITION) and Antifungal Combinations for Treatment of Cryptococcal Meningitis in Africa (ACTA) cohort was determined using univariable logistic regression models with days on ART as the single predictor (using restricted cubic splines with 3 knots) and mortality as the outcome for (*A*) 2-week and (*B*) 10-week mortality, restricted to participants on ART for up to 6 months. The gray shading represents 95% confidence intervals. Mortality risk in those on ART for more than 6 months and those not on ART at the time of cryptococcal meningitis diagnosis are shown for comparison, with error bars representing 95% confidence intervals, and the dotted line indicating the mortality risk estimate in those not on ART.

Mortality risk differed significantly according to time on ART but did not decline progressively with increasing ART duration, with ongoing mortality reductions up to 6 months of prior ART exposure, but increased mortality risk in those on ART for more than 6 months (Figure 2). In models adjusted for antifungal treatment group, participants who had initiated ART within 14 days had higher mortality risk than those on ART for longer (Table 3); 2-week mortality risk was 20.8% (95% CI: 11.5–30.2) in those on ART for ≤14 days, 10.4% (95% CI: 3.6–17.2) on ART for >2 weeks to 2 months, 7.1% (95% CI: 0–14.9) on ART for >2 months to 6 months, and 13.0% (95% CI: 8.5–17.6) in those on ART for >6 months. Using the group not on ART as a reference, those initiating ART within 14 days had a 1.7-fold (95% CI: 1.0–2.8) increased risk of 2-week mortality and a 1.4-fold (95% CI: 1.0–2.0) increased risk of 10-week mortality. Adjustment for age and sex did not change the strength of association between 2- and 10-week mortality and recent ART initiation. Similar mortality trends were seen in the analysis using combined AMBITION and ACTA trial cohorts (Supplementary Table 1 and Figure 2).

### Effect of ART Discontinuation on Mortality

In an analysis restricted to the 72 AMBITION participants who had initiated ART within 14 days of cryptococcal meningitis diagnosis, there was a significant interaction between the clinical decision to interrupt ART at enrollment and ART timing on mortality risk at 2- and 10-weeks (*P* value for interaction .01 for 2-week mortality and .05 for 10-week mortality). Two-week mortality was 35% (8/23) among those who continued ART versus 14% (7/49) among those who discontinued ART (*P* = .06); at 10 weeks’ mortality was 52% (12/23) in those who continued ART versus 31% (15/49) in those who discontinued ART (*P* = .08) (Table 3). Compared to the group not on ART, the risk ratio for 2-week mortality was 3.7 (95% CI: 1.8–7.5) in those who had initiated ART within 14 days of cryptococcal meningitis diagnosis and continued ART, and 0.9 (95% CI: .4–2.1) in those who discontinued ART (*P* = .01).

## DISCUSSION

Individuals started on ART within 2 weeks before presentation with cryptococcal meningitis, presumably in the context of active cryptococcal infection, have higher mortality, whereas those presenting on ART for 2 weeks or more have a more favorable prognosis. Individuals presenting with cryptococcal meningitis after 6 months on ART had similar characteristics and outcomes to individuals not on ART, with self-reported adherence and viral load data indicating that the majority of these individuals were not on effective suppressive HIV therapy.

Previous analyses have found no difference in acute mortality between individuals presenting on and off ART. However, considering all individuals “on ART” as a homogenous group is misleading, encompassing those initiated on ART with active cryptococcal meningitis, individuals presenting with unmasking IRIS after ART initiation in the context of early subclinical cryptococcal infection, individuals who have not yet immune reconstituted, and individuals experiencing ART treatment failure.

Our results confirm the findings of Rhein et al [8] who observed excess 2-week mortality in patients diagnosed with cryptococcal meningitis within 14 days of ART initiation in a Ugandan cohort. Our data showing that the vast majority (91/115, 79%) of individuals diagnosed with cryptococcal meningitis within 14 days of initiating effective ART had symptoms including headache that preceded their ART initiation, strongly suggest that patients had active but undiagnosed cryptococcal meningitis at ART initiation. It is known from prior randomized clinical trials that ART initiation in the context of untreated or inadequately treated cryptococcal meningitis (at the time of presentation or very early during antifungal treatment) is associated with increased mortality [5, 15]; the exact causal mechanisms are unknown, but almost certainly involve immunopathology and excessive dysregulated inflammation from ART-mediated immune restoration in the context of a high pathogen load [16].

A rational approach to reduce excess mortality in individuals presenting with cryptococcal meningitis having very recently started ART may be to interrupt ART. Discontinuation of ART to manage IRIS is generally avoided because of risks of developing HIV drug resistance and other opportunistic infections, and a lack of evidence that ART discontinuation in this situation would lead to a sufficiently rapid reduction in ART-mediated immune restoration to be of benefit [17, 18]. In the context of individuals who initiated ART within 14 days of being diagnosed with cryptococcal meningitis the risk–benefit balance is different; significant ART-mediated immune restoration is unlikely to have occurred within such a short timeframe, making it plausible that ART interruption can prevent pathological inflammatory responses.

An expert opinion published by investigators in the AMBITION trial [3, 19] recommended discontinuation of ART in individuals presenting with cryptococcal meningitis within 2 weeks of ART initiation; continuation of ART in those on ART for longer than 14 days but less than 6 months with reported adherence to ART; and ART discontinuation in individuals who reported poor adherence or were likely to have resistance, with reinitiation of appropriate ART after 4 to 6 weeks of antifungal treatment [11]. We observed lower 2-week mortality in recent ART initiators if ART was interrupted and demonstrated that this approach did not lead to harm. However, this was not a protocol-specified intervention, uptake was variable, and selection biases impacting comparisons of those on ART for less than 2 weeks in whom ART was interrupted at diagnosis of cryptococcal meningitis and those in whom ART was continued preclude any definitive conclusions from our data.

Individuals established on ART for 2 weeks to 6 months had evidence of less severe cryptococcal disease with lower fungal burdens and higher CD4 counts. Excess mortality risk rapidly declined with increasing time on ART at presentation, matching the risk seen in individuals not on ART shortly after 2 weeks, and declining further up to 6 months on ART. Many of these individuals are likely to have had an “unmasking” IRIS, with evidence for more active inflammation in terms of higher CD4 counts, higher CSF white cell counts, and higher CSF protein levels, and meningitis could have been prevented by CD4 testing, reflex cryptococcal antigen screening and preemptive fluconazole at ART initiation [20–23].

People presenting with cryptococcal meningitis after longer durations of ART had similar clinical presentations and outcomes to individuals not on ART, reflecting that the majority of these individuals were not virologically suppressed, and highlighting the frequency of treatment interruptions and cyclical nature of the HIV care cascade [24]. Individuals who are currently “in care” but not suppressed on ART, usually because of difficulties in maintaining adherence to therapy, account for a considerable burden of advanced HIV disease. “Continuing” ART in this setting would in most cases effectively be ART reinitiation in the context of active cryptococcal meningitis; our data therefore support recommendations to hold ART in this patient group until 4 to 6 weeks of antifungal therapy have been completed [25].

The results of this study should be interpreted with consideration of the following limitations. Analyses were limited by the relatively low number of participants who had recently initiated ART and ART interruption in these individuals was not randomized, making it difficult to draw firm conclusions about association between ART interruption and mortality. ART adherence data were subject to recall and desirability bias, and we had incomplete viral load data, resistance testing, or assessment of ART drug levels.

In conclusion, our findings confirm increased risk of early mortality in cryptococcal meningitis patients who present very soon after initiating ART and provide some evidence supporting recommendations to temporarily interrupt ART in these individuals and in those likely to be failing ART after longer durations of therapy, while continuing ART in individuals established on effective therapy [11].

## Supplementary Material

ciae586_Supplementary_Data

## Supplementary Data

Supplementary materials are available at *Clinical Infectious Diseases* online. Consisting of data provided by the authors to benefit the reader, the posted materials are not copyedited and are the sole responsibility of the authors, so questions or comments should be addressed to the corresponding author.

## Note

***Financial support.*** Supported by a grant (TRIA2015-1092) through the European and Developing Countries Clinical Trials Partnership, with assistance from the Swedish International Development Cooperation Agency, as well as by funding from the Department of Health and Social Care, the Foreign Commonwealth and Development Office, the Medical Research Council, and the Wellcome Trust (MR/P006922/1). Funding was also provided by the National Institute for Health and Care Research (NIHR) through a Global Health Research Professorship (RP-2017-08-ST2-012, to J. N. J.) with aid from the U.K. government to support global health research. The views expressed in this article are those of the authors and not necessarily those of the U.K. Department of Health and Social Care or the U.K. Foreign Commonwealth and Development Office. G. M. was supported by the Wellcome Trust (098316, 214321/Z/18/Z, and 203135/Z/16/Z), and the National Research Foundation (South Africa) (Grant No 64787). The funders had no role in the study design, data collection, data analysis, data interpretation, or writing of this report. This research was funded, in part, by the Wellcome Trust. For the purpose of open access, the author has applied a CC BY public copyright licence to any Author Accepted Manuscript version arising from this submission.
