# Supplementary material for: Recent Antiretroviral Therapy Initiation Is Associated With Increased Mortality Risk in HIV–associated Cryptococcal Meningitis: An Analysis of Clinical Trial Data From Africa
Source: Clin Infect Dis. 2025 Jan 15;81(1):75–83. doi: 10.1093/cid/ciae586 (PMC12314494; doi:10.1093/cid/ciae586)
Supplement: ciae586_Supplementary_Data [file ciae586_supplementary_data.docx]

**Supplementary Table 1.** Mortality outcomes among the combined AMBITION and ACTA participants with HIV-associated cryptococcal meningitis stratified by ART status at the time of presentation.

| **ART status** | | **2-week mortality**^*^ | |  | **10-week mortality**^*^ | | |  |
| --- | --- | --- | --- | --- | --- | --- | --- | --- |
|  | | Mortality risk (95% CI) | N | Risk ratio (95% CI) | Mortality risk (95% CI) | N | Risk ratio (95% CI) | |
| A. | |  |  |  |  |  |  | |
| Not on ART | | 15.7% (13.0-18.3) | 111/707 | Base | 30.2% (26.8-33.6) | 214/707 | Base | |
| On ART | Overall | 16.0% (13.4-18.5) | 125/777 | 1.00 (0.9 – 1.3) | 32.5% (29.2-35.8) | 255/777 | 1.06 (0.9 - 1.2) | |
|  | *≤14 days* | *21.5% (14.2-28.8)* | *26/120* | *1.37 (0.9 – 2.0)* | *36.0% (27.6-44.3)* | *44/120* | *1.19 (0.9 – 1.5)* | |
|  | *>14 days ≤2 months* | *13.8% (7.9-19.7)* | *18/130* | *0.88 (0.6 – 1.4)* | *30.8% (22.9-38.7)* | *40/130* | *1.02 (0.8 – 1.3)* | |
|  | *>2 months ≤6 months* | *7.3% (2.1-12.5)* | *7/92* | *0.47 (0.2 – 1.0)* | *23.3% (14.8-31.8)* | *22/92* | *0.77 (0.5 – 1.1)* | |
|  | *>6 months* | *16.7% (12.4-20.9)* | *50/307* | *1.06 (0.8 – 1.4)* | *33.5% (28.2-38.9)* | *101/307* | *1.11 (0.9 – 1.3)* | |
|  | | ART: antiretroviral therapy; CI: confidence interval.  * Mortality risk derived from the generalized linear model adjusted for treatment | | | | | |  |
